# Multiple malignant transformations of an ovarian mature cystic teratoma

**DOI:** 10.3332/ecancer.2020.1009

**Published:** 2020-02-04

**Authors:** Kristen Cagino, Daniel Levitan, Nina Schatz-Siemers, Rasa Zarnegar, Eloise Chapman-Davis, Kevin Holcomb, Melissa K Frey

**Affiliations:** 1 Department of Gynecologic Oncology, New York Presbyterian Hospital/Weill Cornell Medical Center, New York, NY 10021, USA; 2 Department of Pathology and Laboratory Medicine, New York Presbyterian Hospital/Weill Cornell Medical Center, New York, NY 10021, USA; 3 Department of Surgery, New York Presbyterian Hospital/Weill Cornell Medical Center, New York, NY 10021, USA

**Keywords:** mature cystic teratoma, carcinoid syndrome, malignant transformation, struma ovarii, strumal carcinoid

## Abstract

**Background:**

Malignant transformation of mature cystic teratomas (MCTs) is a rare phenomenon. The most common histology of a malignant transformation is squamous cell carcinoma, and there are limited reports of multiple malignancies arising in a single MCT. Further data are necessary to guide management of these atypical cases.

**Case:**

We present the case of a 48-year-old with MCT containing a malignant papillary thyroid carcinoma (PTC) arising in the context of struma ovarii and a carcinoid tumour.

**Conclusion:**

Malignant transformations of MCTs are exceedingly rare with no guidelines on management. We use this case to demonstrate an approach for the workup and management of malignantly transformed MCTs.

## Introduction

Teratomas are germ cell tumours containing several types of tissue derived from at least two of the three germ layers (i.e., endoderm, mesoderm and ectoderm). Most well-sampled teratomas will reveal tissue elements that are derived from the ectoderm. Teratomas can be further classified as mature or immature. Mature teratomas are benign and tend to present as cystic lesions, commonly known as dermoid cysts. On the other hand, immature teratomas are usually more solid and are malignant in nature. Thyroid tissue can be seen in mature cystic teratomas (MCTs). Struma ovarii is a term applied to ovarian neoplasms that are composed entirely or predominantly of thyroid tissue. They are considered monodermal teratomas and account for less than 3% of all MCTs [1]. Although rare, struma ovarii carries the potential for malignant transformation. Another uncommon ovarian neoplasm is carcinoid tumour. Carcinoid tumours can exist as primary ovarian tumours, but they have also been described in association with MCTs or mucinous cystadenomas or as disease metastatic to the ovary from a separate primary site [2]. While primary ovarian carcinoid tumours have been known to metastasize, their malignant potential varies from case-to-case, with the stage of disease playing an important prognostic role. They are best considered low-grade malignancies. Carcinoid tumours fall under the umbrella of neuroendocrine tumours, which have the ability to lead to carcinoid syndrome, a paraneoplastic syndrome manifesting as dermatologic, gastrointestinal, pulmonary and/or circulatory symptoms. We present a case of a pre-menopausal woman with a left adnexal mass found on postoperative pathology to have two separate malignant tumours:

- Papillary thyroid carcinoma (PTC) existing within the context of a struma ovarii.
- A carcinoid tumour.

## Case

A 48-year-old, gravida 4, para 2 woman presented for evaluation of a left adnexal mass. She reported being diagnosed by imaging with a left ovarian MCT approximately 8 years prior. She recently developed sharp lower abdominal pain and abnormal uterine bleeding. Pelvic imaging including a magnetic resonance imaging (MRI) demonstrated a large mixed solid and cystic mass in the anterior pelvis measuring 10 cm × 7.2 cm × 6.0 cm with small fat nodules within the cystic component of the mass, suggestive of a left adnexal teratoma. The patient’s medical history was notable for asthma and diabetes mellitus. Her surgical history was notable only for a prior laparoscopic bilateral tubal ligation. Family history was significant for a paternal grandmother with ovarian cancer.

The patient underwent examination demonstrating mild fullness and tenderness in the right adnexa. She had an office endometrial biopsy due to abnormal uterine bleeding, which demonstrated benign secretory endometrium. The patient underwent a laparoscopic left salpingo-oophorectomy and right salpingectomy. Intra-operative evaluation demonstrated that the left adnexa was replaced by a complex cystic mass with an irregular surface. The cyst was removed intact in an endocatch bag with no evidence of rupture.

**Figure 1.**
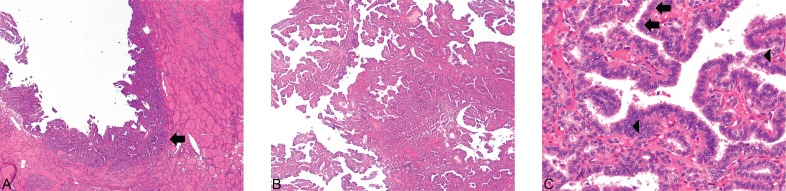
(A) Struma and PTC ×40. (B) PTC ×40. (C) PTC ×400. PTC (arrow) can be seen arising in the background of benign thyroid tissue (1A) (Hematoxylin and Eosin, x40). The carcinoma exhibits the classical papillary architecture (1B) as well as diagnostic cytologic features (Hematoxylin and Eosin, x40). The latter includes cuboidal-to-low columnar cells with overlapping nuclei, nuclear grooves (arrows), and the presence of optically clear chromatin (arrowheads) (1C) (Hematoxylin and Eosin, x400).

**Figure 2.**
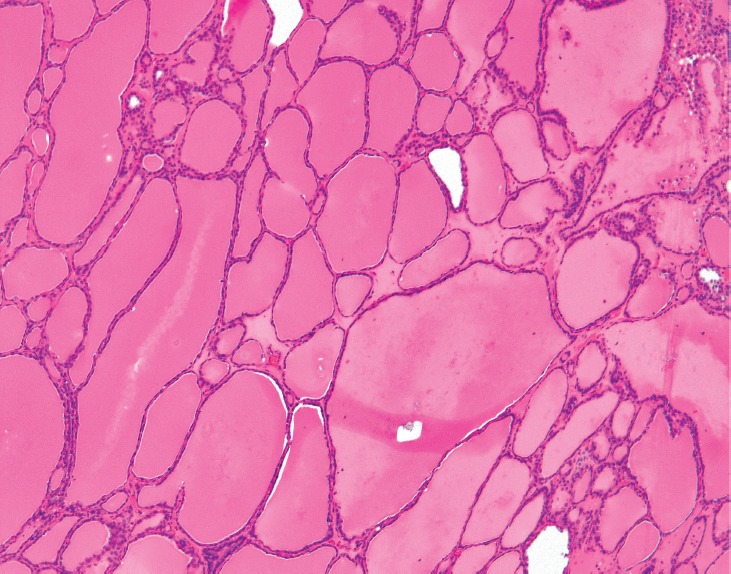
Struma ×100. Also present are foci of relatively more recognizable thyroid tissue, consistent with a struma ovarii. The colloid-filled acini are lined by a single layer of flat-to-low cuboidal cells just like in the eutopic thyroid. (Hematoxylin and Eosin, x100).

**Figure 3.**
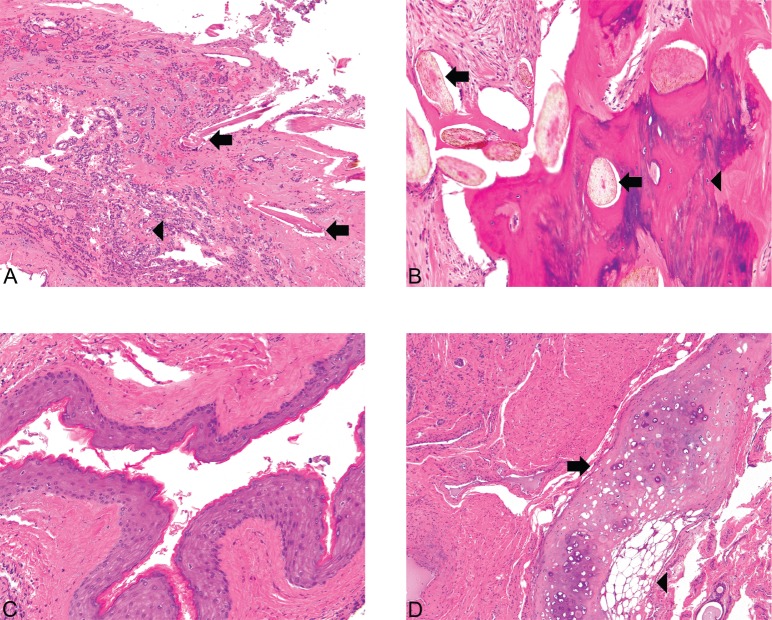
(A): Struma and hair follicles ×100. The mature teratoma shows intimate admixture with thyroid tissue, the greatest component of the tumor in this case. Hair follicles (arrows) can be seen amongst small acini filled with colloid (microfollicular pattern) (arrowhead) (Hematoxylin and Eosin, x100). (B): Bone and hair follicles ×200. (C): Epidermoid ×200. (D) Cartilage ×100. Mature teratoma component. The tumor shows foci consistent with a mature teratoma showing derivation from at least two of the three germ layers (ectoderm, mesoderm, and endoderm). The various mature elements include hair follicles (arrows) intermixed with bone (arrowhead) (3A), squamous epithelium (3C), and cartilage (arrow) juxtaposed with adipose tissue (arrowhead) (3D) [Hematoxylin and Eosin, x200 (1A, 1B), x100 (1C)].

**Figure 4.**
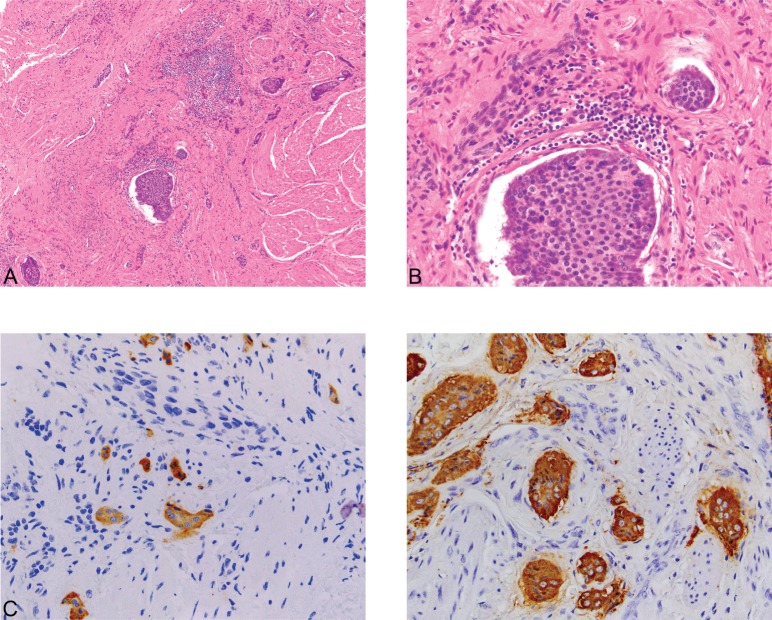
(A): Carcinoid ×100. (B): Carcinoid ×400. (C): Synaptophysin ×400. (D): Chromogranin ×400. An incidental focus of insular carcinoid (0.3 cm) was identified in one of the tumor sections. The carcinoid tumor consists of solid nests made up of uniform cells with centrally-placed nuclei (4A) (Hematoxylin and Eosin, x100) and (4B) (Hematoxylin and Eosin, x400). The tumor is positive for the neuroendocrine immunohistochemical markers synaptophysin (4C) and chromogranin A (4D) (x400), while negative for CK7 (not shown). The Ki-67 proliferation index of the tumor is less than 1%, and no mitotic figures were identified (not shown).

Histopathologic examination of the left ovary and fallopian tube revealed PTC arising in a struma ovarii. The PTC showed prominent papillary architecture with epithelium composed of cuboidal cells, which exhibited cellular crowding and nuclear overlap. Occasional nuclei showed optically clear cytoplasm, while others had nuclear grooves (Figure 1). Of note, the struma ovarii made up the bulk of the tumour and consisted of colloid-filled acini lined by a single layer of flat-to-low cuboidal cells like those seen in normal thyroid tissue (Figure 2). However, other teratomatous elements were also identified, including mature bone, cartilage, adipose tissue, squamous epithelium and hair follicles (Figure 3). Microscopic evaluation of the submitted tissue also revealed a small carcinoid tumour measuring 0.3 cm. Immunohistochemistry was performed and showed that the carcinoid tumour was positive for the neuroendocrine markers synaptophysin and chromogranin A (Figure 4), while negative for TTF-1 and CK7. The adjacent struma ovarii was positive for TTF-1 and CK7. The right fallopian tube showed benign paratubal cysts.

After multidisciplinary discussion at both endocrinology and gynaecologic oncology tumour boards, the patient was advised to undergo thyroid/neck sonogram and laboratory tests. The latter would include thyroid function tests as well as an assessment of serum thyroglobulin and chromogranin A levels. Ultrasound revealed punctate 1–2 mm colloid cysts in the left thyroid gland but was otherwise unremarkable. The patient had elevated serum thyroglobulin of 59.4 ng/mL; all other lab values were within normal limits. Given that the patient had no evidence of carcinoid syndrome, no biomarkers (serotonin, catecholamines and histamine) were sent. Follow-up sonogram demonstrated a right ovarian mass and the patient was recommended hysterectomy and right oophorectomy. The patient underwent hysterectomy and right oophorectomy without issue, and all pathology was negative for malignancy. The patient has been disease-free for 6 months and is planned for close surveillance.

## Discussion

MCTs are the most common germ cell tumours of the ovary, constituting approximately 20% of ovarian neoplasms. They are bilateral in 8%–14% of cases [1]. MCTs commonly occur in women of reproductive age, but have also been found in postmenopausal women and children. Most patients with MCTs are asymptomatic, but they may also present with abdominal pain, a palpable abdominal mass or vaginal bleeding. Management of MCTs is provider-dependent and based on the individual patient, although surgery is often warranted. If surgery is chosen, options for removal include cystectomy versus oophorectomy or salpingo-oophorectomy. At the time of surgery, intraoperative pathology is often recommended in the event that there is a malignant component. The risk of malignant transformation is relatively rare and estimated to occur in 0.17%–2% of cases [2]. Most cases of malignant transformation are in the form of squamous cell carcinoma. However, less commonly reported malignancies include thyroid carcinoma, which may arise in the setting of a struma ovarii, and carcinoid tumours.

MCTs have been reported to contain thyroid tissue in 5%–20% of cases. Struma ovarii is a rare form of MCT in which thyroid tissue is the predominant component (>50% of the tumour). Struma ovarii occurs in approximately 5% of ovarian teratomas. Malignant struma ovarii is rare and has an incidence of 0.1%–0.3% [3]. The most common type of malignancy associated with struma ovarii is classic PTC, followed by follicular thyroid carcinoma, follicular variant of papillary carcinoma and highly differentiated follicular carcinoma of ovarian origin (HDFCO). HDFCO is a relatively new entity described by Roth and Karseladze [3] and characterised by extraovarian dissemination of thyroid elements that histologically resemble normal thyroid tissue. Metastasis is relatively rare and occurs in 5%–23% of cases [4]. Debate exists regarding the optimal management of malignant struma ovarii.

While surgery is the mainstay of treatment, surgical options vary from ovarian cystectomy to unilateral salpingo-oophorectomy, bilateral salpingo-oophorectomy, total abdominal hysterectomy/bilateral salpingo-oophorectomy and full staging. Additional adjuvant treatment options include radiation, chemotherapy, thyroidectomy, radioactive iodine and thyroid suppression. Some sources recommend post-operative iodine scans to assess for residual disease and trending serum thyroglobulin to monitor for recurrence [5]. Recurrence rates range from 15% to 38% with an average time to recurrence of 4 years [2]. In a 2002 review of 24 patients, 8 received adjuvant therapy with thyroidectomy and radioactive iodine while 16 did not receive adjuvant therapy. One of the patients experienced persistent disease postoperatively, but all other patients had a complete response to initial surgery. All eight patients who recurred had not received adjuvant therapy. No recurrences were found in a group of seven patients who received adjuvant therapy after complete response to initial surgery [6]. This suggests that adjuvant therapy may be necessary for preventing recurrence.

Primary ovarian carcinoid tumours are relatively rare tumours which resemble well-differentiated neuroendocrine tumours of the gastrointestinal tract. They make up less than 0.1% of ovarian tumours [7]. Carcinoid tumours can occur independently or in conjunction with MCTs or mucinous cystadenomas. There are four main histopathologic categories of carcinoid tumours: insular, trabecular, strumal and mucinous. Insular is the most common type and is associated with carcinoid syndrome, which commonly manifests as flushing, sweating and diarrhoea. Strumal carcinoid is the second most common subtype, and is a combination of thyroid and carcinoid tissue. It is not generally associated with carcinoid syndrome; instead, it is often seen with virilism and postoperative thyroid hormone imbalances such as thyroid storm and hypothyroidism. Sixty percent of strumal carcinoids arise in MCTs or solid teratomas. Trabecular and mucinous carcinoid tumours are seen less frequently. The histopathologic features of insular carcinoids as described in the fourth edition of the ‘World Health Organization’s Classification of Tumours of Female Reproductive Organs’ include small acini and solid nests of uniform, polygonal cells with round, centrally located nuclei. Trabecular carcinoid is composed of elongated wavy ribbons of cells. A strumal carcinoid is the combination of an insular or trabecular carcinoid with a struma ovarii. Strumal carcinoids often have intestinal-type mucinous glands. Last, mucinous carcinoid shows acini lined by columnar or cuboidal epithelium with goblet cells. The various types of primary ovarian carcinoid tumour seem to have unique immunohistochemical profiles. While insular and trabecular carcinoids are CK7 positive and CK20 negative, mucinous carcinoids are usually CK7 negative and CK20 positive. Strumal carcinoids usually express TTF-1 and CK7 in the fibrous stroma but not in the carcinoid tumour cells [8]. Similar to malignant strumal ovarii, there is debate regarding the correct management of carcinoid tumours, although surgical excision is often the first line. Again, treatment ranges from cystectomy to hysterectomy with bilateral salpingo-oophorectomy. Metastatic disease often results from the spread of either the carcinoid or thyroid component, seldom both. Metastatic thyroid disease can be treated with thyroidectomy and radioactive iodine. Thiotepa (an alkylating chemotherapeutic) has been used for the treatment of metastatic carcinoid tumour. Carcinoid tumours have the potential to lead to carcinoid syndrome, a neuroendocrine paraneoplastic syndrome, in approximately 30%–40% of well-differentiated neuroendocrine tumours. Carcinoid syndrome typically occurs in tumours arising from the midgut but has also been described with bronchial and pancreatic carcinoids. Patients with strumal carcinoids are less likely to present with carcinoid syndrome. Carcinoid syndrome develops due to the secretion of several substances including serotonin, histamine, kallikrein, prostaglandins and tachykinins. Effects of carcinoid syndrome include facial flushing, hypotension, diarrhoea, tachycardia and bronchoconstriction [9].

## Conclusion

MCTs are the most common germ cell tumour of the ovary, but malignant transformation is exceedingly rare. Although most patients are asymptomatic at the time of diagnosis, abdominal pain, a palpable mass and vaginal bleeding may be presenting symptoms. This is a rare case demonstrating the occurrence of multiple malignant transformations within a single MCT found after left salpingo-oophorectomy for a mass that appeared benign on pre-operative evaluation. No guidelines exist for the diagnosis and management of these lesions, so we offer a multimodal approach. Workup can involve ultrasound, MRI and salpingo-oophorectomy. Our patient subsequently underwent hysterectomy and oophorectomy, but other surgical options include ovarian cystectomy, oophorectomy, unilateral or bilateral salpingo-oophorectomy and Total Abdominal Hysterectomy/Bilateral Salpingo-oophorectomy with full staging. Adjuvant treatment for struma ovarii includes Radiation therapy, chemotherapy, thyroidectomy, Radioactive Iodine and thyroid suppression, and for carcinoid tumours includes thyroidectomy, RAI and thiotepa for metastatic lesions. Management of multiple lesions likely involves a combination of the above options. The overall goals of management are palliation of symptoms (if any), preventing recurrence or spread of disease and preservation of fertility (if possible).

## Conflicts of interest

Kristen Cagino, Daniel Levitan, Nina Schatz-Siemers and Rasa Zarnegar have nothing to disclose.

## Funding statement

Melissa K Frey has research support by Invitae.
